# Gene Editing of the Decoy Receptor *LeEIX1* Increases Host Receptivity to *Trichoderma* Bio-Control

**DOI:** 10.3389/ffunb.2021.678840

**Published:** 2021-06-21

**Authors:** Meirav Leibman-Markus, Rupali Gupta, Lorena Pizarro, Ofir Gershony, Dalia Rav-David, Yigal Elad, Maya Bar

**Affiliations:** Department of Plant Pathology and Weed Research, Plant Protection Institute, Agricultural Research Organization, Volcani Institute, Rishon LeZion, Israel

**Keywords:** xylanase, *LeEIX1*, *Trichoderma*, bio-control, tomato, gene-editing

## Abstract

Fungal and bacterial pathogens generate devastating diseases and cause significant tomato crop losses worldwide. Due to chemical pesticides harming the environment and human health, alternative disease control strategies, including microorganismal bio-control agents (BCAs), are increasingly sought-after in agriculture. Bio-control microorganisms such as *Trichoderma* spp. have been shown to activate induced systemic resistance (ISR) in the host. However, examples of highly active bio-control microorganisms in agricultural settings are still lacking, due primarily to inconsistency in bio-control efficacy, often leading to widespread disease prior to the required ISR induction in the host. As part of its plant colonization strategy, *Trichoderma* spp. can secrete various compounds and molecules, which can effect host priming/ISR. One of these molecules synthesized and secreted from several species of *Trichoderma* is the family 11 xylanase enzyme known as ethylene inducing xylanase, EIX. EIX acts as an ISR elicitor in specific plant species and varieties. The response to EIX in tobacco and tomato cultivars is controlled by a single dominant locus, termed LeEIX, which contains two receptors, *LeEIX1* and *LeEIX2*, both belonging to a class of leucine-rich repeat cell-surface glycoproteins. Both receptors are able to bind EIX, however, while LeEIX2 mediates plant defense responses, LeEIX1 acts as a decoy receptor and attenuates EIX induced immune signaling of the LeEIX2 receptor. By mutating *LeEIX1* using CRISPR/Cas9, here, we report an enhancement of receptivity to *T. harzianum* mediated ISR and disease bio-control in tomato.

## Introduction

Plant pathogens are the foremost yield limiting factor for many crops in open field and greenhouse cultivation systems (Vitti et al., 2015), and this trend is expected to increase due to climate and regulatory changes. Fungal and bacterial pathogens are the cause of significant tomato crop losses worldwide, generating devastating diseases, in some cases due to wide host range, relatively limited information on pathogen biology and infection strategies, and their ability to remain quiescent for long periods of time and become virulent upon changing conditions (He et al., 2016). Pesticidal strategies can lack effectivity and are often a source of pollution and detrimental effects to consumer health (Yadav and Devi, 2017; Leong et al., 2020), with many pesticides becoming increasingly banned worldwide.

Bio-control of foliar diseases is a potential alternative, non-toxic means of management of foliar pathogens. Induced resistance has been documented as one of the mechanisms responsible for bio-control. Induced resistance is recognized as an important mode of action to achieve bio-control in vegetative tissues (Sequeira, 1983; Kuc, 1987). Induced systemic resistance (ISR) effected by various microorganisms can protect plants against pathogens (Paulitz and Matta, 2000).

One of the most studied commercial bio-control agents (BCAs) is isolate T39 of *Trichoderma harzianum*, which serves as a model for commercial bio-control and the mechanisms involved. T39 has been shown under commercial greenhouse conditions to control foliar pathogens including *Botrytis cinerea, Pseudoperonospora cubensis, Sclerotinia sclerotiorum* and *Sphaerotheca fusca* (syn. *S. fuliginea*) (Elad, 2000a,b).

ISR is examined by applying a BCA distal to the plant organ subsequently challenged by a pathogen. It was thus demonstrated that T39 causes ISR, inducing plant defense against *B. cinerea* in several plant hosts. T39 was applied to the soil or the lower leaves, with the pathogen being subsequently applied to the upper canopy of the plants. Given the spatial separation of T39 application from the pathogens, disease protection was attributed to ISR imparted by *T. harzianum* T39 (De Meyer et al., 1998).

Given the lack of examples of highly successful BCAs in practical disease management, it is possible that current BCAs are unlikely to be able to rapidly achieve satisfactory and stable disease control. The characteristics of BCAs are such that slight changes in the external environment could potentially result in drastic changes in the system dynamics and hence bio-control efficacies (Juroszek and von Tiedemann, 2011). This may explain often observed inconsistencies in bio-control efficacy in practice, as spatio-temporal environmental heterogeneity is a rule rather than an exception, and can lead to widespread disease prior to the required colonization or ISR induction by the bio-control agent. Generating increased receptivity in the host to immunity activation by BCAs could potentially improve BCA effectivity and disease control.

As part of its plant colonization strategy, *Trichoderma* spp. can secrete various antimicrobial compounds and molecules, which can effect host priming/ISR. One of these molecules synthesized by, and secreted from several species of *Trichoderma*, including *T. viride* and *T. reseii*, is a family 11 xylanase enzyme known as ethylene inducing xylanase, EIX. The *Trichoderma* fungal protein elicitor EIX, induces ethylene biosynthesis, electrolyte leakage, expression of PR proteins and the hypersensitive response (HR) in specific plant species and/or varieties (Bailey et al., 1990; Sharon et al., 1992; Ron et al., 2000; Elbaz et al., 2002). EIX was shown to specifically bind to the plasma membrane of responsive cultivars of both tomato and tobacco (Hanania and Avni, 1997). The response to EIX in tobacco and tomato cultivars is controlled by a single dominant locus, termed LeEIX (Ron and Avni, 2004). The LeEIX locus contains two receptors, *LeEIX1* and *LeEIX2*, both belonging to a class of leucine-rich repeat cell-surface glycoproteins. Both receptors are able to bind the EIX elicitor, while only the LeEIX2 receptor mediates plant defense responses (Ron and Avni, 2004). LeEIX1 acts as a decoy receptor and attenuates EIX induced signaling of the LeEIX2 receptor (Bar et al., 2010, 2011).

EIX acts to increase plant immunity through binding and downstream signaling of LeEIX2 (Ron and Avni, 2004), while LeEIX1 acts to block this immunity promoting downstream signaling (Bar et al., 2010, 2011). We show here that removing LeEIX1 resulted in stronger immune activation, leading to increased host response to *Trichoderma* bio-control agents and enhancement of disease resistance conferred by *Trichoderma*.

## Results

### Generation of *LeEIX1* CRISPR Mutants

**Figure 1:**
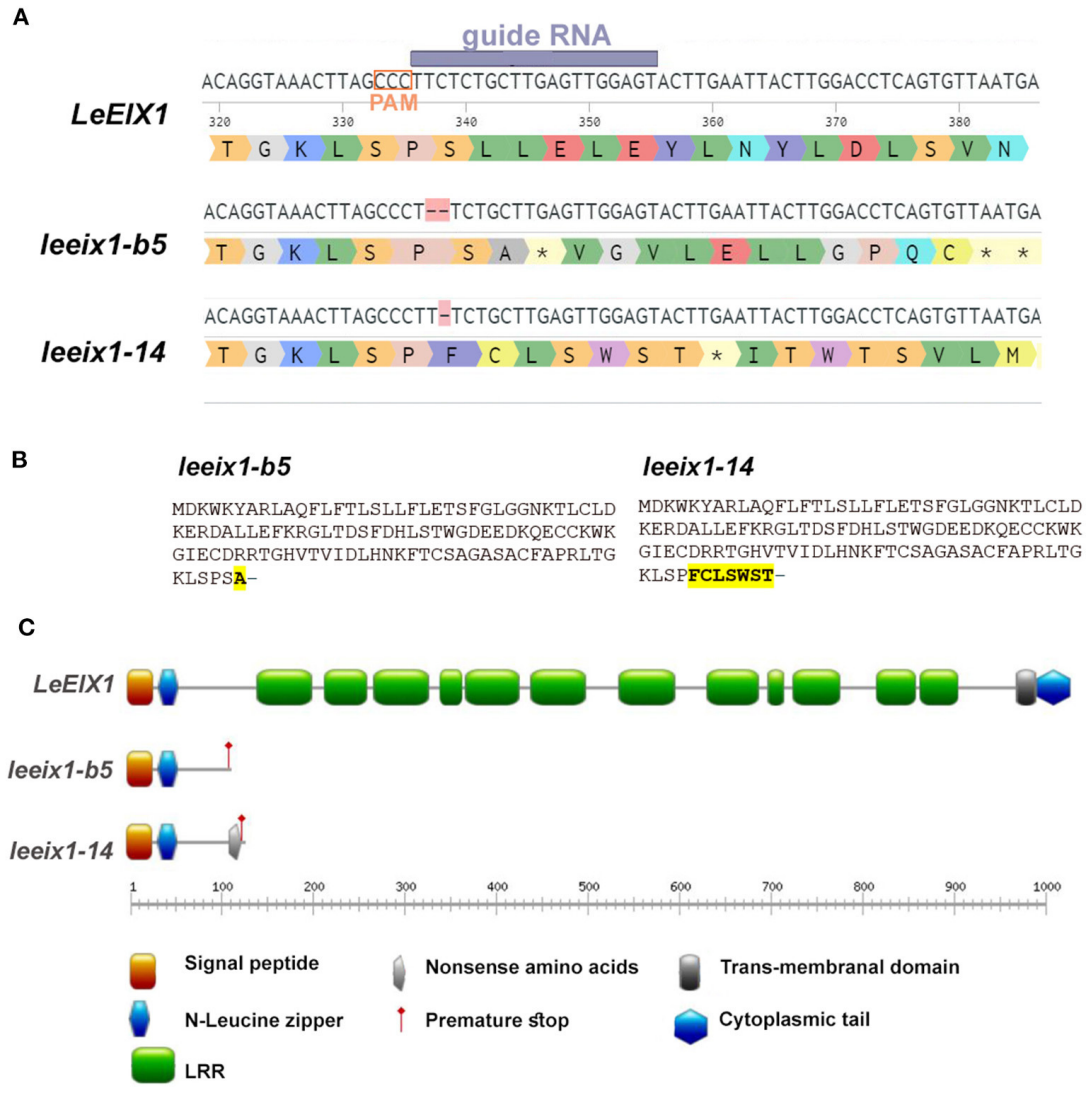
Description of generated *leeix1* lines. **(A)** Representation of gRNA/ PAM site and resultant mutations in the *LeEIX1* sequence. **(B)** Predicted protein sequence of the mutants. **(C)** Protein domain analysis of LeEIX1 alongside the mutant truncated proteins. **(A)** Generated with Benchling (Biology Software) (2021). Retrieved from https://benchling.com. **(C)** Generated with Prosite MyDomain (Hulo et al., 2008).

Previous research has indicated that LeEIX1 attenuates defense signaling in *Solanaceae* in a BAK dependent manner (Bar et al., 2010, 2011). To examine this phenomenon, we generated *LeEIX1* knockouts using CRISPR/Cas9 as detailed in the Materials and Methods section. CRISPR/Cas9 is a versatile, design-easy, and low-cost tool, which has been used efficiently for precise genome editing in plants. Since its emergence several years ago, CRISPR technology has revolutionized gene editing in molecular biology and agricultural contexts (Molla et al., 2020). Designing specific gRNAs to target only *LeEIX1* and not *LeEIX2*, we were able to obtain two independent homozygous lines, one with a two base deletion (line b5) and the other with a one base deletion (line 14), at a PAM site ~330 nucleotides after the LeEIX1 start codon (Figure 1A, Supplementary Figure 1). Both mutations are predicted to result in a frame shift causing a stop codon, resulting in a truncated 113 amino acid protein from the N-terminus of the 1031 amino acid full LeEIX1, with *leeix1-14* additionally having 7 “nonsense” amino acids prior to the premature stop (Figure 1B). The truncated proteins formed in the mutants contain only the signal peptide and N-terminal Leucine zipper, and do not have the LRR domains important for ligand recognition and protein-protein interactions, or the transmembranal domain required for PM insertion (Figure 1C). Thus, these truncated proteins formed would likely not be inserted in the membrane, or be able to bind the xylanase ligand or protein interactors- leading to null of LeEIX1 functionality.

### Verification of Agricultural Quality of Generated Mutants

**Figure 2:**
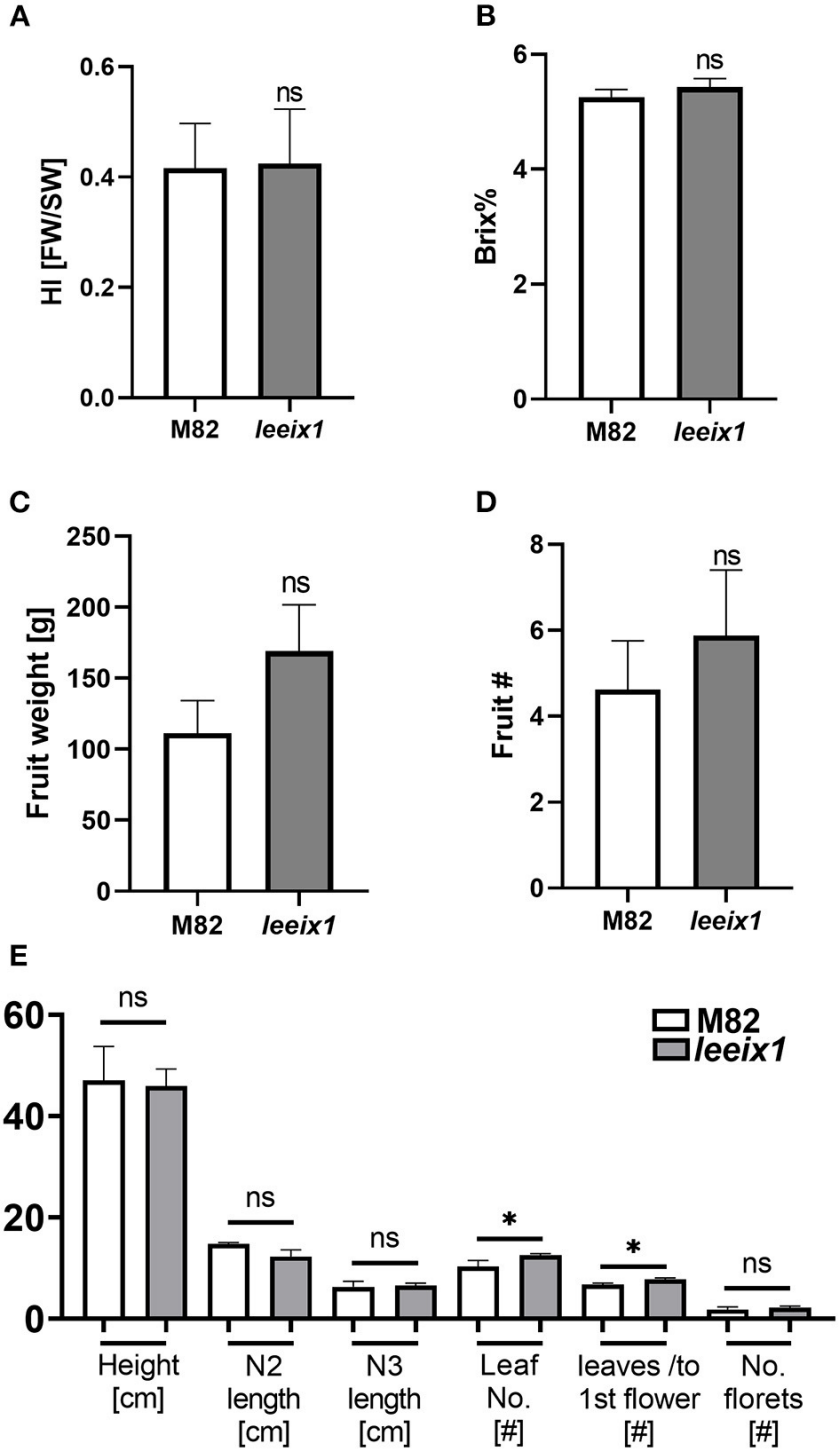
*leeix1* mutants have similar agricultural quality as the background M82 line. Agricultural and developmental parameters were measured in M82 and *leeix1* plants. Both mutant lines, #b5 and #14, were used in the experiments. **(A)** Harvest index (HI) of plants was calculated as the ratio between the total mass of fruit yield and the total biomass. **(B)** Total soluble sugars were measured using a refractometer and are expressed as °Brix. **(C)** Total fruit weight per plant. **(D)** The average total number of tomato fruits produced per plant. **(E)** Analysis of growth and development parameters: Height, length of nodes 2 and 3, Number of leaves, Number of leaves produced in the vegetative stage (before the first flower), and Number of florets. Average ± SEM of at least four independent replicates is shown, *N* = 18. Except in the number of leaves, no statistically significant differences were observed among WT and *leeix1* (*t*-test, Welch's correction). *p < 0.05.

*LeEIX1* and *LeEIX2* were originally identified using a screen of the *Solanum pennellii* M82 introgression populations (Eshed et al., 1992; Ron and Avni, 2004). Based on our previous knowledge that the introgression line IL-7-5 has ~350 genes on the short arm of chromosome 7 originating from *S. pennellii*, including both *LeEIX1* and *LeEIX2* which contain various mutations, has ostensibly normal development, we surmised that the mutated *LeEIX1* lines would likely not have developmental defects. We analyzed their growth and developmental quality, finding that the *leeix1* plants have similar developmental progression, agricultural quality, yield, and tomato quality, as their wild type M82 background line (Figure 2, Supplementary Figures 2, 3). Figure 2 shows results obtained from both homozygous lines, used simultaneously in experiments, while supplemental Supplementary Figure 2 provides data for each line individually.

### *T. harzianum* Treatment Results in Stronger Disease Reduction in *leeix1* Mutants

**Figure 3:**
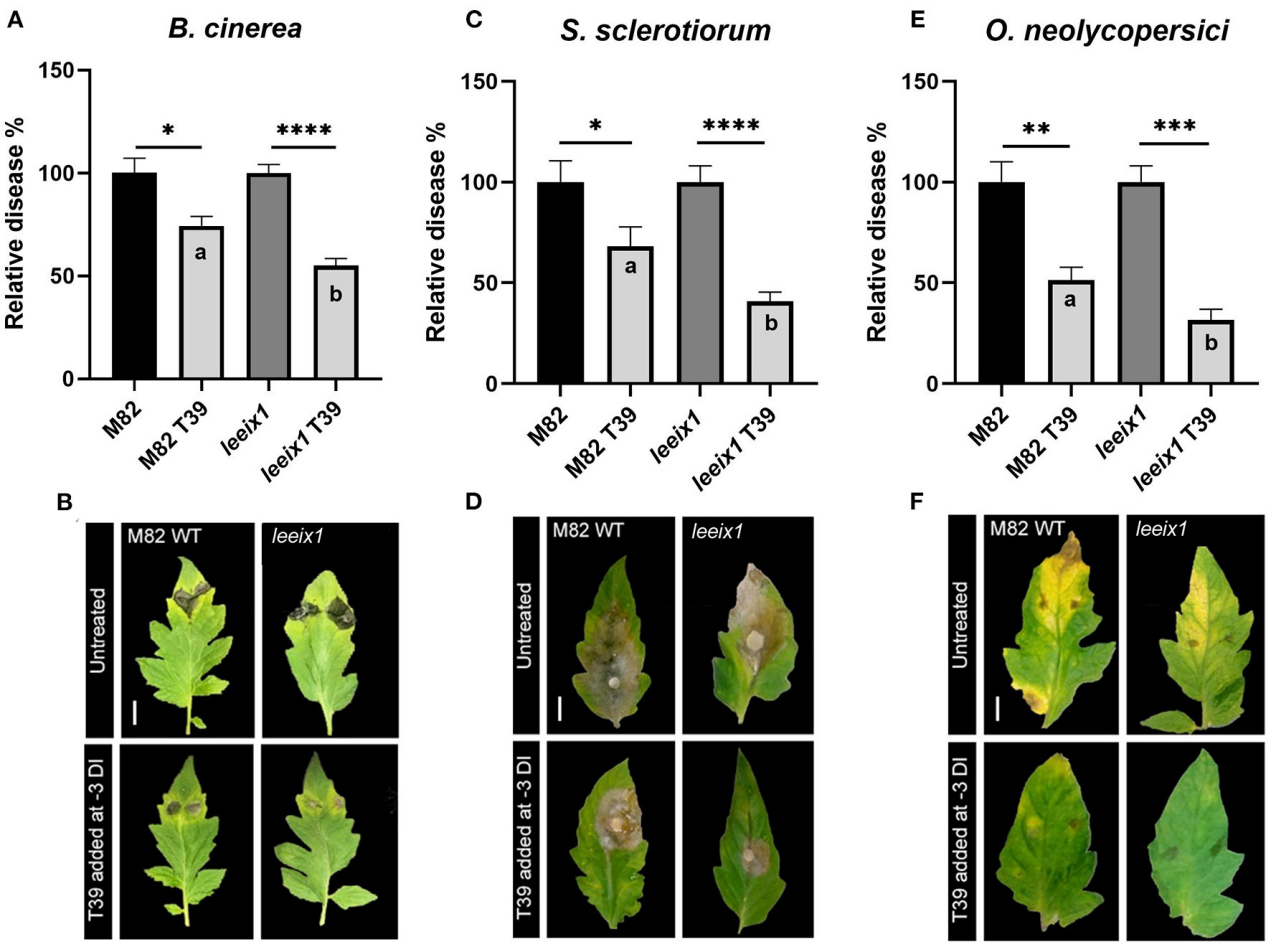
*leeix1* mutants have improved disease resistance in response to *T. harzianum* T39. WT M82 and *leeix1* plants were challenged with *B. cinerea* (10^6^ spores /mL) **(A,B)**, *S. sclerotiorum* (mycelial plugs from 5 day old plates) **(C,D)**, and *O. neolycopersici* (10^4^ conidia /mL) **(E,F)**. Relative disease area was calculated as the lesion area measured 5 days after inoculation in each genotype treated with T39 as compared to mock-treated controls for *B. cinerea* and *S. sclerotiorum*, and similarly, 10 days after inoculation, for *O. neolycopersici*. **(B,D,F)** representative images taken on day of measurement. **(A)** Average ± SEM of 6 independent replicates is shown, *N* = 85. **(C)** Average ± SEM of 3 independent replicates is shown, *N* = 20. **(E)** Average ± SEM of 3 independent replicates is shown, *N* = 12. In all cases, asterisks represent statistical significance of T39 treatment over control in a one way analysis of variance with a Bonferroni *post-hoc* test, *p* < 0.0165, and letters represent statistical significance between the WT and *leeix1* T39 treated samples in a one way analysis of variance with a Bonferroni *post-hoc* test, *p* < 0.03.

To examine whether *T. harzianum* mediated bio-control was improved in *leeix1* mutants, we treated WT and *leeix1* plants with *T. harzianum* T39, and infected the plants with the necrotrophic fungi *B. cinerea* or *S. sclerotiorum*, or the biotrophic fungus *Oidium neolycopersici*, 3 days after the first treatment. *leeix1* plants displayed greater reduction in disease levels following T39 treatment than WT plants (Figure 3, Supplementary Figure 4).

### *T. harzianum* Treatment Elicits Stronger Defense Response Activation in *leeix1* Mutants

**Figure 4:**
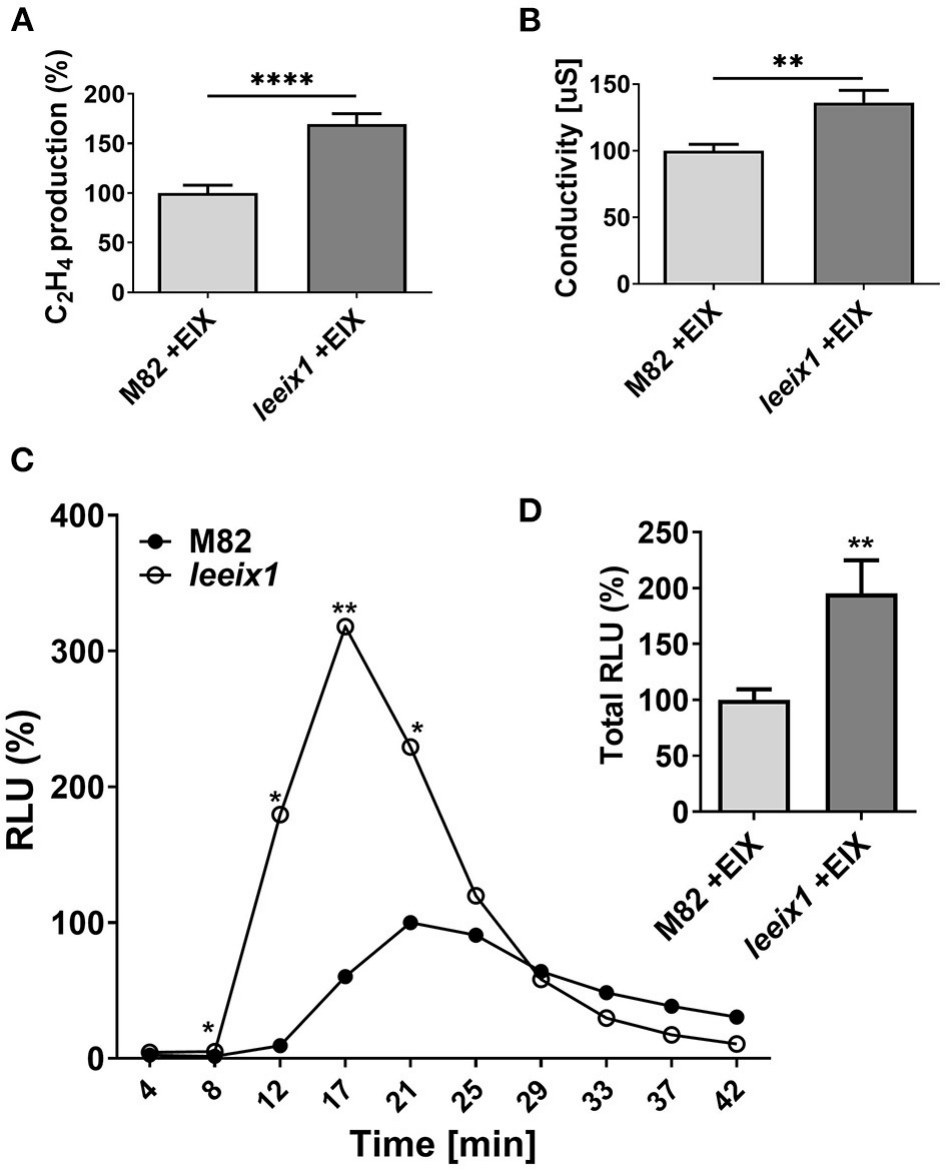
*leeix1* mutants have improved defense responses to the *Trichoderma* elicitor Xyn11/EIX. **(A)** Ethylene induction after EIX elicitation in M82 and *leeix1* was measured using gas-chromatography. M82 average ethylene production was defined as 100%. Average ± SEM of 3 independent experiments is presented, *N* = 10. Asterisks represent statistical significance in a *t*-test with Welch's correction (****p < 0.0001). **(B)** Conductivity of M82 and *leeix1* samples immersed in water supplemented with EIX for 24 h was measured. Average ± SEM of 3 independent replicates is shown. Asterisks represent statistical significance in *t*-test with Welch's correction (**p < 0.01). **(C)** ROS production of WT M82 and *leeix1* was measured immediately after EIX application every 4 min, using the HRP-luminol method. Average ± SEM of 3 independent experiments is shown, *N* = 26 per time point. Asterisks represent statistical significance in multiple *t*-tests (one per time point), *p < 0.05, **p < 0.01. **(D)** Shows total ROS production, **p < 0.01 in a *t*-test with Welch's correction, *N* = 26.

As *T. harzianum* primed enhanced disease resistance in *leeix1* mutants, we examined whether *leeix1* mutants had stronger immune responses than control plants when treated with *Trichoderma*. We measured defense responses following treatment with the *Trichoderma* derived elicitor EIX, in *leeix1* mutants and control plants. *leeix1* plants displayed greater responses to EIX, generating significantly higher levels of ethylene (Figure 4A) and ion leakage (Figure 4B), as well as reactive oxygen species (ROS, Figures 4C,D) in response to EIX treatment.

### *leeix1* Possesses Higher Receptivity to Additional Trichoderma Strains

**Figure 5:**
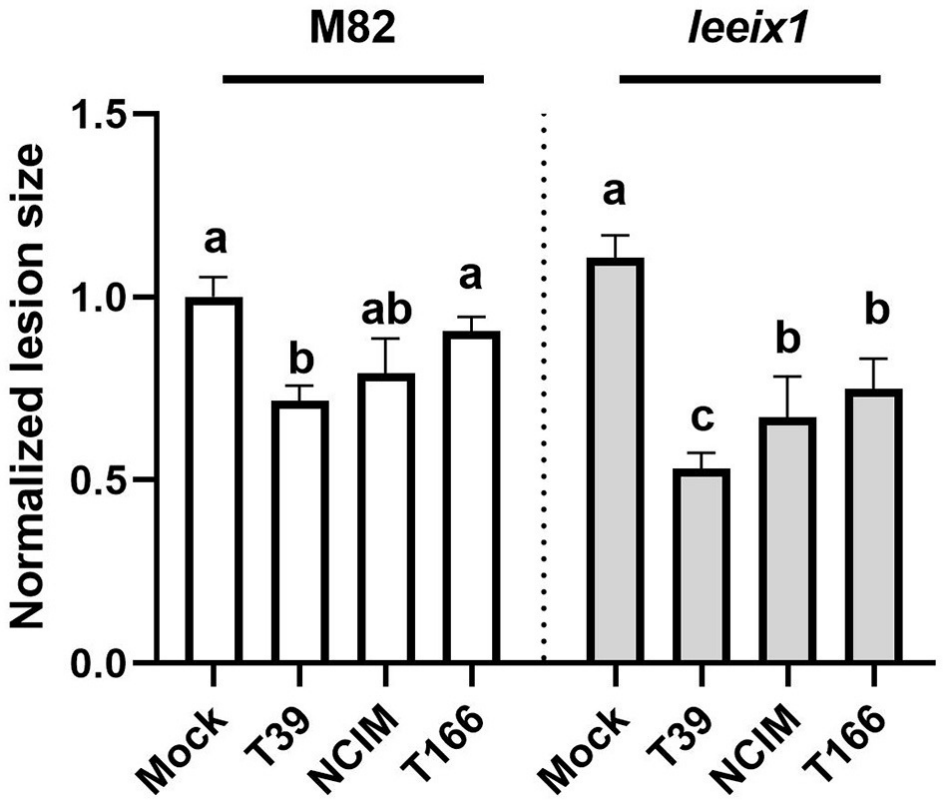
*leeix1* mutants have improved disease resistance in response to additional *Trichoderma* strains. WT M82 and *leeix1* plants were challenged with *B. cinerea* (10^6^ conidia /mL). Relative disease area was calculated as the lesion area measured 5 days after inoculation in each genotype treated with indicated *Trichoderma* strains as compared to mock-treated controls. Average ± SEM of 4 independent replicates is shown, *N* = 16, *p* < 0.0077 in a two-tailed *t*-test.

To further characterize the improved receptivity to bio-control we observed in *leeix1* mutants, we examined two more *Trichoderma* isolates in their ability to induce resistance. Another isolate of *T. harzianum*, NCIM1185 (hereinafter: NCIM), was selected, as well as an isolate of a different species of *Trichoderma, T. longibrachiatum*, known as T166. Both isolates have proven bio-control activity, though neither was previously tested in tomato (Kapat et al., 1998; Elad and Kapat, 1999; Maymon et al., 2004). Similarly to the *T. harzianum* T39 strain, the two other *Trichoderma* isolates showed improved bio-control activity against *B. cinerea* in *leeix1* as compared with the WT M82 background (Figure 5). These results indicate a common mode of action for the *Trichoderma* isolates.

### Trichoderma Leaf Colonization Is Similar in WT and *leeix1* Mutants

**Figure 6:**
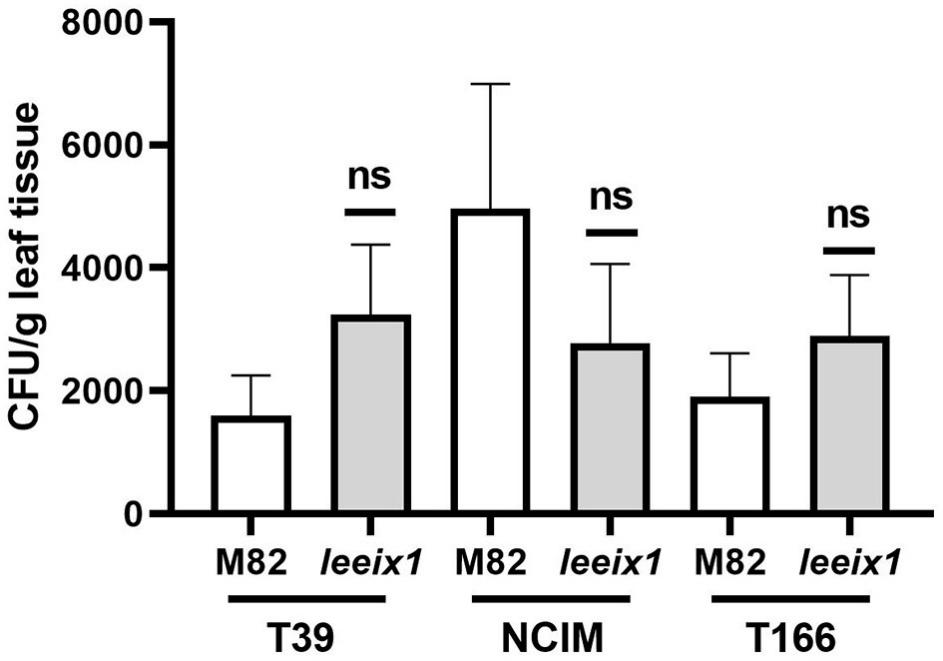
*Trichoderma* colonizes WT and *Leeix1* leaves to similar levels. CFU amount of indicated *Trichoderma* strains in leaf tissue. *Trichoderma* spp. were cultured on potato dextrose agar (PDA) (Difco Lab) plates and incubated at 25°C for 7 days. Conidia concentration was adjusted to 10^6^ cells mL^−1^, and sprayed onto plants. Two weeks after treatment, leaves were harvested and washed in water with thorough shaking. Leaves were then removed and sterilized in bleach for 2 min, then ground with a mortar and pestle. One gram of the ground tissue was re-suspended in 1 mL water. Serial dilutions were plated on PDA plates containing 0.02% of rose bengal. Resultant colonies were counted to determine CFU count in the leaves. Graph represents average CFU per leaf ± SEM of three independent experiments, *N* = 8. No significant differences among samples were found (two-tailed *t*-test).

Micro-organismal bio-control of plant diseases can be a result of induced systemic resistance, as was reported previously in several cases including for T39 (De Meyer et al., 1998; Elad, 2000a; Perazzolli et al., 2008; Meller-Harel et al., 2014), or, alternatively, can also be the result of microorganism colonization of the plant and direct or chemical effects of said colonizing microorganisms on the attacking pathogens (De Meyer et al., 1998; Elad, 2000a; Perazzolli et al., 2008; Meller-Harel et al., 2014). Additionally, BCA colonization of the plant may be a requirement for the induction of ISR. We examined colonization of different *Trichoderma* strains in tomato leaves, finding no difference in colonization between WT and *leeix1* (Figure 6). As *Trichoderma* colonization is not favored in *leeix1*, we conclude that ISR enhancement in *leeix1* is likely to be due to another mechanism.

## Discussion

BCAs have promising prospects for use as an alternative to fertilizers and pesticides, allowing for the development of environmentally friendly agricultural practices. To this end, microbial BCAs have been intensely investigated in recent years (Koch et al., 2018; Tian et al., 2020). Generally, the activities of microbial BCAs are varied and can serve to promote plant vigor, or assist the plant in combating pathogens, either directly or indirectly. Exemplary BCA activities include nitrogen fixation (Li et al., 2017), phosphate solubilization (Mitra et al., 2020), secretion of metabolite-like compounds which can directly control phytopathogens (Kenawy et al., 2019), and induction of host systemic resistance that allows the plant to better combat invading pathogens (Nguvo and Gao, 2019). Various BCAs, including *Trichoderma* spp., are known to be systemic inducers of plant immunity (Gupta and Bar, 2020).

*Trichoderma* spp. are opportunistic plant colonizers that trigger host ISR and elicit rapid plant defense responses (De Meyer et al., 1998; Nawrocka and Małolepsza, 2013). Root and leaf colonization by *Trichoderma* can prime plant defenses, resulting in robust plant responses to subsequent pathogen attack (Elad and Kirshner, 1993; Gupta and Bar, 2020). *Trichoderma* spp. are potent inducers of plant ISR (Alfiky and Weisskopf, 2021).

In past years, the use of *Trichoderma* spp. in both research and agriculture has seen significant increases. *Trichoderma* spp. are interesting models to investigate when probing the interaction between plant hosts and their symbionts, with proven and anticipated successes as BCAs for the promotion of sustainable agriculture (López-Bucio et al., 2015; Guzmán-Guzmán et al., 2017). *Trichoderma* interacts with many plant hosts, and can thus play significant roles in both plant growth and plant resistance to pathogens in varied ecosystems (Kashyap et al., 2017). Not surprisingly, efforts are underway to understand the molecular mechanisms that influence the ability of *Trichoderma* to effect ISR and promote plant disease resistance.

Although BCAs have become extremely attractive agriculturally, many of them have not become agricultural market products, because their activity in promoting plant growth and/or pathogen resistance is insufficient or irreproducible under changing environments, or affected by unknown factors (Mathre et al., 1999; Harman et al., 2012). To address this issue, this work utilized genome editing to genetically manipulate the tomato host to be more receptive to *Trichoderma* activity.

Several MAMPs from *Trichoderma* have been reported to be involved in ISR induction (Hoitink et al., 2006; Vinale et al., 2008; Hermosa et al., 2012). *Trichoderma* spp. produced enzymes including cellulase and xylanase, are known to stimulate resistance in plants, independent of their enzymatic activity (Martinez et al., 2001). The first discovered and perhaps most extensively studied *Trichoderma* MAMP is EIX, which elicits plant defenses in responsive tomato and tobacco cultivars, independent of its enzymatic activity (Furman-Matarasso et al., 1999; Hanania et al., 1999; Rotblat et al., 2002). EIX induces 1-aminocyclopropane-1-carboxylic acid (ACC) synthase expression, ethylene biosynthesis, and ISR (Matarasso et al., 2005). The xylanase EIX is recognized by the transmembranal LRR pattern recognition receptors (PRRs) LeEIX1 and LeEIX2 (Ron and Avni, 2004; Bar and Avni, 2009).

Previous work has demonstrated that LeEIX1 serves as a decoy receptor, attenuating the signals propagated by LeEIX2, toward immune response activation (Bar et al., 2010, 2011). Here, we report that CRISPR/Cas9 knockout plants of *LeEIX1* showed improved disease reduction (Figures 3, 5, Supplementary Figure 4) and immune response activation (Figure 4) following *Trichoderma* treatment. *LeEIX1* knockout, as expected, did not affect agricultural quality in greenhouse settings (Figure 2, Supplementary Figures 2, 3).

*Trichoderma* colonization leads to transcriptional and translational changes in plant host tissues (Shoresh et al., 2010; Hermosa et al., 2012). It has been suggested that the stimulation of plant growth and activation of ISR requires *Trichoderma* to colonize the host plant (Nawrocka and Małolepsza, 2013). *Trichoderma* species are able to colonize different plant organs, altering plant metabolism by producing growth-stimulating compounds (Elad, 2000a; Harman et al., 2004). Some *Trichoderma* strains promote plant fitness by enhancing nutrient availability as well as resistance against various pathogens (Howell, 2003; Harman, 2006). However, it has been reported that bio-control potential might not be dependent on the level of *Trichoderma* colonization, but rather, on the activation of systemic resistance as a result of the initial colonization signals (Ruano-Rosa et al., 2016), as well as host immunity inducing molecules (MAMPs) secreted by *Trichoderma* (Ramírez-Valdespino et al., 2019). Indeed, we observed that *T. harzianum* T39 and NCIM, and *T. longibrachiatum* T166 were able to colonize the leaves of both M82 and *leeix1* mutant plants to a similar extent. There were no significant differences in the amount of *Trichoderma* found in these plants (Figure 6), supporting the notion that although colonization may be required for ISR activation and *Trichoderma* based priming, differential colonization is not required for achieving differential ISR levels and disease resistance outputs.

In summary, we generated LeEIX1 CRISPR/Cas9 mutants, which retained agricultural quality in greenhouse settings, and investigated their response to *Trichoderma* based bio-control, finding increased disease resistance and defense responses in *leeix1* mutants when compared with WT M82 plants, with no observed differences in *Trichoderma* plant colonization. It will be interesting to examine the performance of these plants and their response to BCAs in the future in agricultural settings.

## Materials and Methods

### Generation of CAS9 *leeix1* Mutants

Tomato CRISPR-Cas9 editing was executed according to Xie et al. (2015). A sgRNA targeting the first exon of *LeEIX1* (nucleotides 355-335 from the atg) was designed using CRISPR-P 2.0 (Liu et al., 2017), taking care not to target *LeEIX2*, which does not contain the PAM present in *LeEIX1* is its sequence. The sgRNA was divided in two, with both oligos being amplified using an sgRNA spacer primer (sgRNA-F 5′-taggtctccCTCAAGCAGAGAAttttagagctagaaat-3′, sgRNA-R 5′-taggtctccTGAGTTGGAGTtgcaccagccgggaa-3′) and terminal specific primers containing a FokI site (L5AD5/L3AD5; see Supplementary Table 1). The products were digested with FokI, and the fragment inserted into a BsaI digested modified pUC57-cloning vector, containing a U6 promoter. The cassette was then subcloned into the binary vector pMR286 (Mily Ron plasmid collection, unpublished). *Agrobacterium tumefaciens* strain GV3101 harboring pMR286 containing the *LeEIX1* sgRNA was used to transform *Solanum lycopersicum* cv M82, according to standard practice (McCormick, 1991). Genomic DNA was extracted from obtained transformant plants, and used as a template for *LeEIX1* sgRNA flanking-fragment amplification. PCR fragments were verified to originate from edited individuals by sequencing (see genotyping primers in Supplementary Table 1). Relevant transgenic lines were selfed, and the resulting T2 plants were re-analyzed. Leaves 4–5 of 6-week-old plants were used for assays.

### Plant Materials and Growth Conditions

*S. lycopersicum* cv M82 and homozygous T3 *leeix1* independent CRISPR lines *leeix1-14* and *leeix1-b5* were grown from seeds in soil (Green Mix; EvenAri, Ashdod, Israel) in a growth chamber, under long-day conditions (16 h:8 h, light:dark) at 24°C. Plants from both independent CRISPR lines were used in assays.

### Fungal Infection

*B. cinerea* isolate *BcI16* and *S. sclerotiorum* isolate *Scl5* were cultured on potato dextrose agar (PDA) (Difco Lab) plates and incubated at 22 ± 4°C for 5–7 days. *B. cinerea* conidia were harvested in 1 mg ml^−1^ glucose and 1 mg ml^−1^ K_2_HPO_4_ and filtered through cheesecloth. Conidia concentration was adjusted to 10^6^ cells ml^−1^ using a haemocytometer. Leaves 4–6 from 5 to 6-week old tomato plants were excised and immediately placed in humid chambers. Each tomato leaflet was inoculated with two droplets of 10 μL suspension. For *S. sclerotiorum*, uniform mycelial plugs (5 mm diameter) were taken using a cork-borer from colony margins and placed mycelial side down on the adaxial surface of each leaf. Inoculated leaves were kept in a humid growth chamber at 21°C.

*O. neolycopersici* was isolated from young leaves of 4–6 week old tomato plants grown in a greenhouse during the winter. Conidia were collected by rinsing infected leaves with sterile water. The concentrations of conidial suspensions were determined under a light microscope using a hemocytometer. Suspensions were adjusted to 10^4^ ml^−1^, and sprayed onto 5–6-week old tomato plants (5 ml per plant). Suspensions were sprayed within 10–15 min of initial conidia collection, with a hand-held spray bottle, and plants were left to dry in an open greenhouse for up to 30 min. Inoculated plants were kept in a humid growth chamber at 21°C. In all cases, controls consisted of leaves treated with water/buffer without the inoculation of pathogen. The diameter of the necrotic lesions or % of infected leaf tissue was measured 3–10 days post inoculation, as indicated, using ImageJ.

### Oxidative Burst (ROS) Measurement

ROS measurement was performed as previously described (Leibman-Markus et al., 2017). 0.5 cm diameter leaf disks were harvested from leaves 4 to 6 of 5–6 week old M82 and *leeix1* plants. Disks were floated in a white 96-well plate (SPL Life Sciences, Korea) containing 250 μl distilled water for 4–6 h at room temperature. The water was then removed, and a ROS measurement reaction containing either 1 μg /mL EIX or water (mock) was added. Light emission was immediately measured using a luminometer (GloMax® Discover, Promega, USA). EIX was purified as previously described (Anand et al., 2021).

### Ethylene Measurement

Ethylene production was measured as previously described (Leibman-Markus et al., 2017). 0.9 cm diameter leaf disks were harvested from leaves 4 to 6 of 5–6 week old M82 and *leeix1* tomato plants. Disks were washed in water for 1–2 h. Every six disks were sealed in a 10 mL flask containing 1 ml assay medium with 1 μg /mL EIX for 5 h at room temperature. Ethylene production was measured by gas chromatography (Varian 3350, Varian, California, USA).

### Electrolyte Leakage Measurement

0.9 cm diameter leaf disks were harvested from leaves 4 to 6 of 5–6 week old M82 and *leeix1* plants. Disks were washed in 50 mL water for 3 h. For each sample, five disks were floated in a 12-well plate containing 1 mL of water with 1 μg /mL EIX, adaxial surface down, at room temperature, with agitation (75 rpm). Conductivity was measured in the water solution after 24 h incubation using a conductivity meter (EUTECH instrument con510).

### Development and Growth Measurements

Growth measurements were performed as previously described by Gur et al. (2010). Plant vegetative weight was determined by excising the plant at the base, after harvesting the fruits. Total fruit yield per plant included both the red and the green fruits. Concentration of total soluble sugars (BRIX percentage) was measured using a digital refractometer with a range of BRIX 0–85 ± 0.2%, from a random sample of 5 red fruits per plant. Harvest index (HI) was calculated as the ratio between the total yield and total biomass.

### *Trichoderma* Treatment and Colonization Assay

*T. harzianum* isolates were cultured on potato dextrose agar (PDA) (Difco Lab) plates and incubated at 25°C for 5–7 days. Spores were harvested in water and filtered through cheesecloth. Conidia concentration was adjusted to 10^6^ cells ml^−1^ using a hemocytometer. Conidia suspensions were sprayed onto plants of desired genotypes. For fungal infections, plants were infected as described above, 3 days after *Trichoderma* treatment. For colonization assays, 2 weeks after *Trichoderma* treatment leaves were harvested and washed in water with thorough shaking. Leaves were then removed and sterilized in bleach for 2 min, and ground with a mortar and pestle. One gram of the ground tissue was re-suspended in 1 mL water. Serial dilutions were plated on PDA plates containing 0.02% of rose bengal dye as a selective agent (Elad et al., 1981). Resultant colonies were counted to determine CFU count in the leaves.

### Statistics and Reproducibility

All data are presented as average ± SEM. Differences between two groups were analyzed for statistical significance using a two tailed *t*-test with Welch's correction for unequal variances, or Holm-Sidak correction for multiple t-tests, where applicable. Differences among three groups or more were analyzed for statistical significance using one-way ANOVA. Regular ANOVA was used for groups with equal variances, and Welch's ANOVA for groups with unequal variances. When a significant result for a group in an ANOVA was returned, significance in differences between the means of different samples in the group were assessed using a *post-hoc* test. The Tukey test was employed for samples with equal variances when the mean of each sample was compared to the mean of every other sample. The Bonferroni test was employed for samples with equal variances when the mean of each sample was compared to the mean of a control sample. The Dunnett test was employed for samples with unequal variances. All statistical analyses were conducted using Prism8™. All experiments were conducted in at least three biologically independent repeats. The number of replicates is indicated for each experiment in each figure legend.

## Data Availability Statement

The datasets presented in this study can be found in online repositories. The names of the repository/repositories and accession number(s) can be found in the article/Supplementary Material.

## Author Contributions

YE and MB: conceptualization and funding. ML-M, RG, LP, OG, and DR-D: experimentation. ML-M, RG, YE, and MB: analysis. ML-M, RG, LP, YE, and MB: manuscript. All authors contributed to the article and approved the submitted version.

## Conflict of Interest

The authors declare that the research was conducted in the absence of any commercial or financial relationships that could be construed as a potential conflict of interest.
