# Supplementary material for: Gene Editing of the Decoy Receptor LeEIX1 Increases Host Receptivity to Trichoderma Bio-Control
Source: Front Fungal Biol. 2021 Jun 21;2:678840. doi: 10.3389/ffunb.2021.678840 (PMC10512410; doi:10.3389/ffunb.2021.678840)
Supplement: Supplementary file 1 [file Data_Sheet_1.pdf]

Supplementary materials

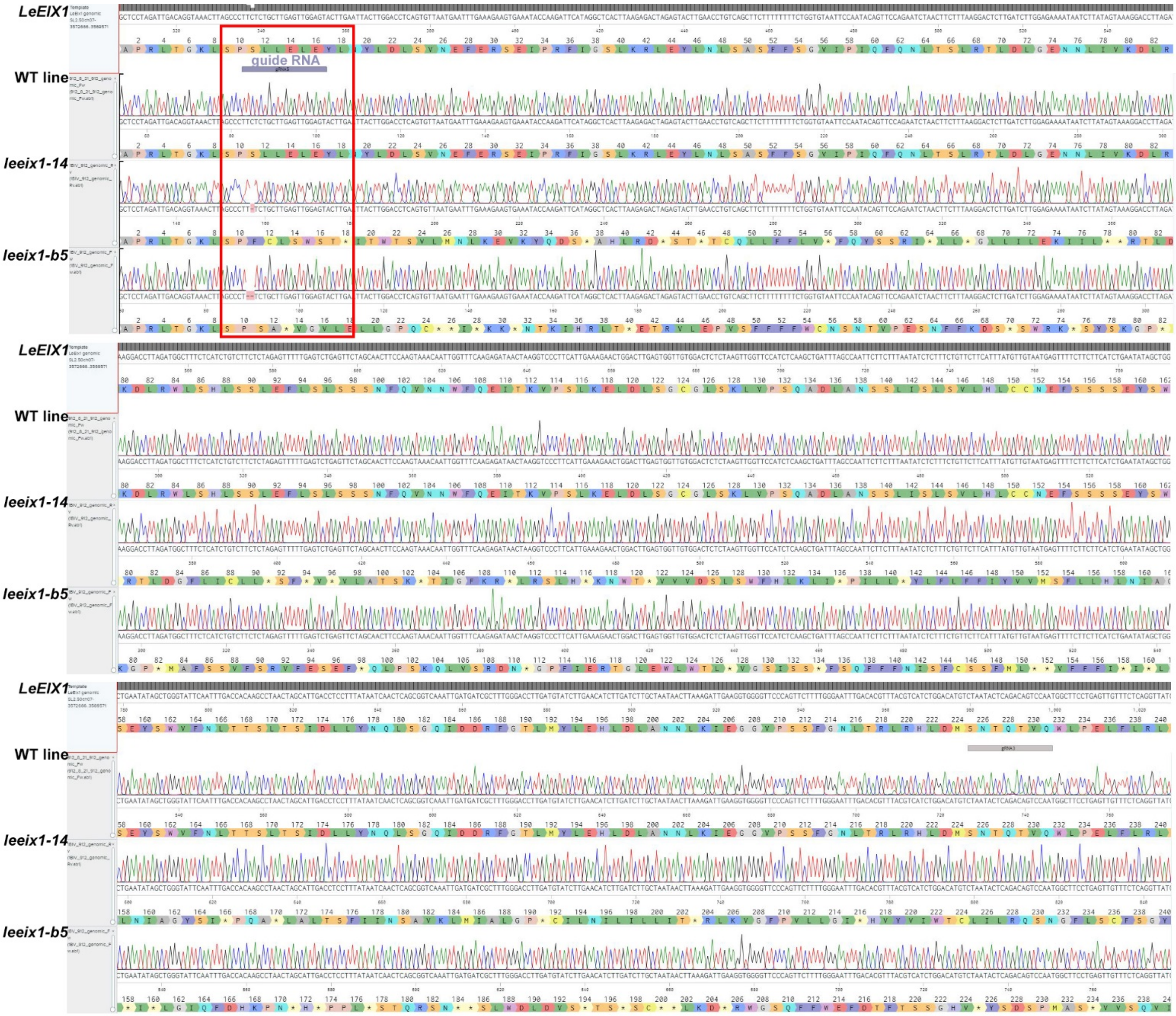

Figure S1: Sequencing of generated *leeix1* lines

Representation of gRNA/ PAM site and resultant mutations in the *LeEIX1* sequence, including sequencing chromatograms above each mutant's resultant protein translation, and a WT line sequenced for reference.

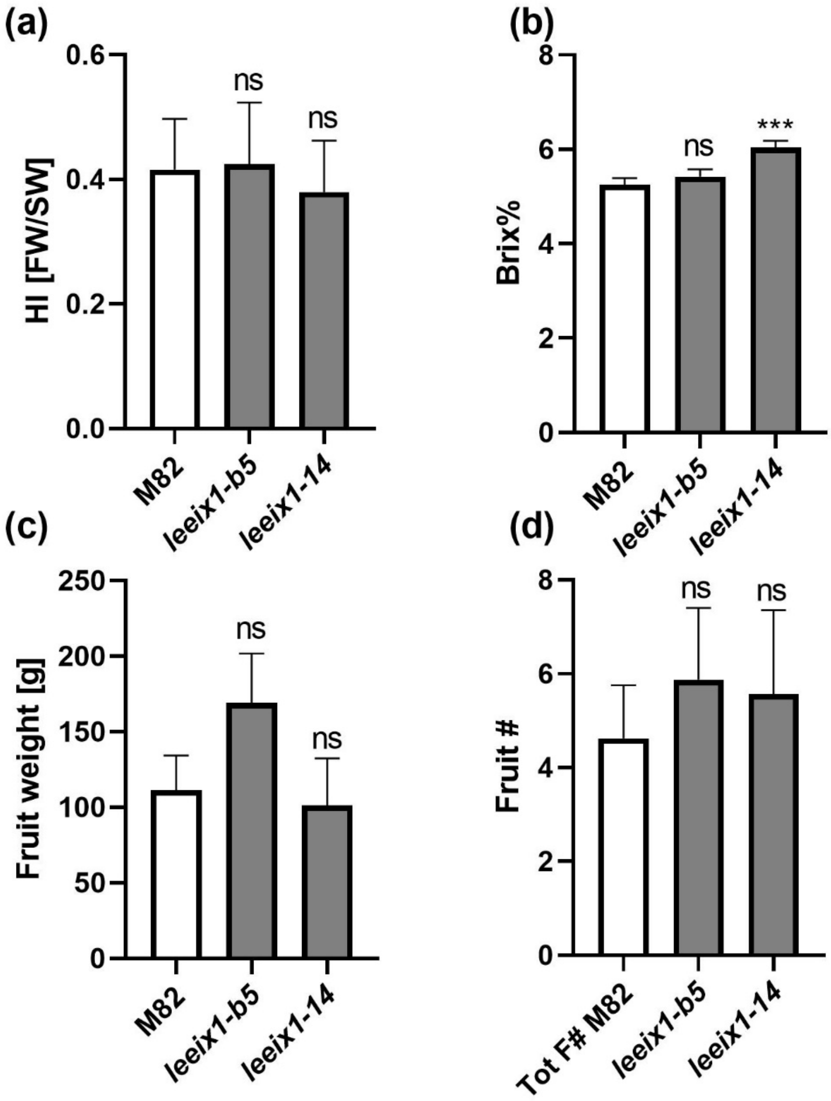

Figure S2: *leeix1* mutants have similar agricultural quality as the background M82 line-separate analysis of two independent *leeix1* mutant lines

Agricultural and developmental parameters were measured in M82 and *leeix1* plants of lines *14* and *b5*. **(a)** Harvest index (HI) of plants was calculated as the ratio between the total mass of fruit yield and the total biomass. **(b)** Total soluble sugars were measured using a refractometer and are expressed as °Brix. **(c)** Total fruit weight per plant. **(d)** The average total number of tomato fruits produced per plant. Average ±SEM of at least three independent replicates is shown, N=8. No statistically significant differences were observed among WT and *leeix1* lines except in the case of Brix, where line 1-4 had increased soluble sugars (t-test, Welch's correction, ***p<0.001). ns= not significant.

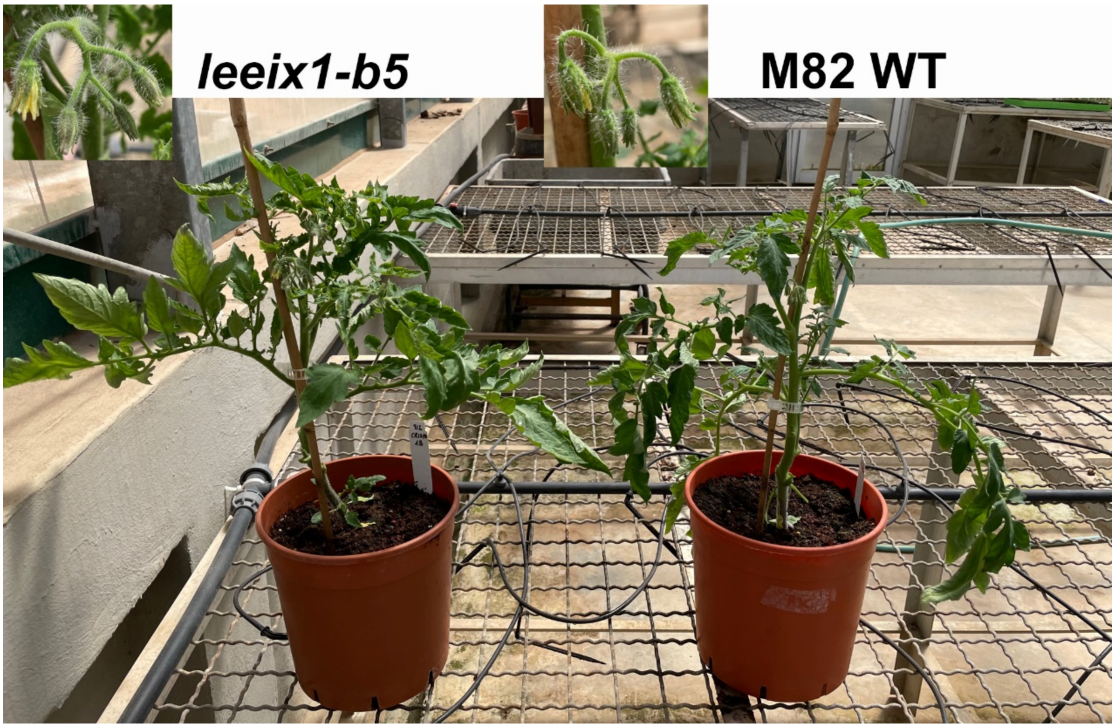

Figure S3: *leeix1* mutants have similar development as the background M82 line.

Representative pictures of 40 day old plants and first inflorescence.

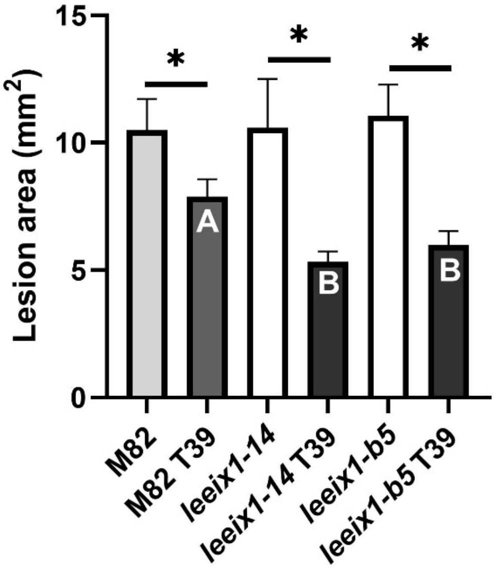

Figure S4: *leeix1* mutants have improved *B. cinerea* disease resistance in response to *T. harzianum* T39 - separate analysis of two independent *leeix1* mutant lines

WT M82 and *leeix1* plants of lines *1-4* and *1b5* were challenged with *B. cinerea* ($10X^6$ spores /mL). Lesion area was measured 5 days after inoculation in each genotype treated with T39 as compared to mock-treated controls. Average ±SEM of 3 independent replicates is shown, N=7. Asterisks represent statistical significance of T39 treatment over control, and letters represent statistical significance between the WT and *leeix1* T39 treated samples, in unpaired two-tailed ttests ($p<0.05$). No significant difference between the two *leeix1* mutant lines was observed.

Supplemental Table 1: Primer pairs used in this work.

Locus	Name	Forward	Reverse
Solyc07g008620	LeEix1gRNA	taggtctccCTCAAGCAGAGAA ttttagagctagaaat	taggtctccTGAGTTGGAGT tgcaccagccgggaa
LTAD5	tRNA-gRNA Cas9 plasmid	L5AD5: CGGGTCTCAGGCAGGATG GGCAGTCTGGGCAACAAA GCAC CAGTGG	L3AD5: TAGGTCTCCAAACGGATGAGC GACAGCAAACAAAAAAAAAA GCACCGACTCG
S5AD5	tRNA-gRNA Cas9 plasmid	CGGGTCTCAGGCAGGATGG GCAGTCTGGGCA	TAGGTCTCCAAACGGATGA GCGACAGCAAAC
Solyc07g008620	LeEix1 Genotyping	GTGACAGAAGAACAGGTC ATGTAACTG	CGATACCTTGTGGTATCCTC CCTC
